# Cumulative Small Effect Genetic Markers and the Risk of Colorectal Cancer in Poland, Estonia, Lithuania, and Latvia

**DOI:** 10.1155/2015/204089

**Published:** 2015-05-26

**Authors:** Pablo Serrano-Fernandez, Dagmara Dymerska, Grzegorz Kurzawski, Róża Derkacz, Tatiana Sobieszczańska, Zbigniew Banaszkiewicz, Hanno Roomere, Eneli Oitmaa, Andres Metspalu, Ramūnas Janavičius, Pavel Elsakov, Mindaugas Razumas, Kestutis Petrulis, Arvīds Irmejs, Edvīns Miklaševičs, Rodney J. Scott, Jan Lubiński

**Affiliations:** ^1^Hereditary Cancer Center, Department of genetics and Pathology, Pomeranian Medical University, Ulica Polabska 4, 70-115 Szczecin, Poland; ^2^Department of Surgery, Collegium Medicum, Nicolaus Copernicus University, Ulica Jagiellonska 13-15, 85-067 Bydgoszcz, Poland; ^3^Asper Biotech, Vaksali 17a, 50410 Tartu, Estonia; ^4^The Estonian Academy of Sciences, Kohtu 6, 10130 Tallinn, Estonia; ^5^Institute of Molecular and Cell Biology, University of Tartu, Riia 23, 23b-134, 51010 Tartu, Estonia; ^6^Estonian Genome Centre of University of Tartu, Riia 23, 23b-134, 51010 Tartu, Estonia; ^7^Department of Molecular and Regenerative Medicine, Hematology, Oncology and Transfusion Medicine Center, Vilnius University Hospital Santariskiu Clinics, Santariškiu g. 2, LT-08661 Vilnius, Lithuania; ^8^State Research Institute, Innovative Medicine Center, Zygimantu 9, LT-01102 Vilnius, Lithuania; ^9^Vilnius Maternity Hospital, Santariškiu g. 2, LT-08661 Vilnius, Lithuania; ^10^Institute of Oncology, Vilnius University, 21/27 M. K. Ciurlionio, LT-03101 Vilnius, Lithuania; ^11^Hereditary Cancer Center, Stradins University, Dzirciema 16 (Pardaugava), Riga LV-1007, Latvia; ^12^Pauls Stradins Clinical University Hospital, Pilsonu 13, Riga LV-1002, Latvia; ^13^Discipline of Medical Genetics, Faculty of Health, University of Newcastle and the Hunter Medical Research Institute, University Drive, Callaghan, NSW 2308, Australia; ^14^Division of Genetics, Hunter Area Pathology Service, John Hunter Hospital, Locked Bag 1000, New Lambton, NSW 2305, Australia

## Abstract

The continued identification of new low-penetrance genetic variants for colorectal cancer (CRC) raises the question of their potential cumulative effect among compound carriers. We focused on 6 SNPs (rs380284, rs4464148, rs4779584, rs4939827, rs6983267, and rs10795668), already described as risk markers, and tested their possible independent and combined contribution to CRC predisposition. *Material and Methods.* DNA was collected and genotyped from 2330 unselected consecutive CRC cases and controls from Estonia (166 cases and controls), Latvia (81 cases and controls), Lithuania (123 cases and controls), and Poland (795 cases and controls). *Results.* Beyond individual effects, the analysis revealed statistically significant linear cumulative effects for these 6 markers for all samples except of the Latvian one (corrected *P* value = 0.018 for the Estonian, corrected *P* value = 0.0034 for the Lithuanian, and corrected *P* value = 0.0076 for the Polish sample). *Conclusions.* The significant linear cumulative effects demonstrated here support the idea of using sets of low-risk markers for delimiting new groups with high-risk of CRC in clinical practice that are not carriers of the usual CRC high-risk markers.

## 1. Introduction

Colorectal cancer (CRC) is one of the most frequent cancers diagnosed in the Polish population and it is the second when listed by mortality in men and third in women [1, 2]. From all newly diagnosed CRC cases, only up to 10% is caused by a high-risk genetic predisposition [3]. Thus, a large proportion of genetic predisposition to CRC may be due to low-penetrance variants. However, while high-risk genes are generally well identified, still little is known about low-risk CRC susceptibility genes. Several studies have led to the identification of genetic markers with odds ratio (OR) ~2 [4–7], although some results are inconclusive and clinical relevance of low-risk markers cannot be definitely established [8–11].

In this study we genotyped 6 SNPs (rs380284, rs4464148, rs4779584, rs4939827, rs6983267, and rs10795668) among nonselected consecutive CRC cases and controls from Estonia, Latvia, Lithuania, and Poland to identify variants and cumulative sets of variants associated with colon cancer risk and to assess potential differences or similarities between these neighboring populations.

These low-risk susceptibility markers included in this study had been previously reported in genome-wide association studies (GWAS) as being related to CRC risk: rs10795668 (10p14); rs3802842 (11q23); rs4779584 (15q13); rs4464148 (18q21); rs4939827 (18q21); and rs6983267 (8q24) [12, 13].

We analyzed the effect of each of those markers separately. But, assuming that small effect genetic markers may have a cumulative effect on compound carriers, we also tried to establish a potential set of markers that could account, in combination, for a high risk of CRC. A recent article signed by Dunlop et al. [14] successfully showed how cumulative effects of low-risk markers can be explored for CRC in several populations. However, cumulative effects of the markers which are object of the present study have not yet been analyzed. Here we follow a similar approach for a smaller number of genetic markers, including the size of the pool of potential risk markers as an additional variable.

## 2. Material

Four groups of patients were included in this study.

 Group 1 consisted of DNA from 166 consecutive CRC Estonian patients registered at the DNA bank of the University of Tartu and Asper Biotech (mean age of diagnosis: 72 years). 166 healthy controls were matched according to gender, age, and ethnic origin.
 Group 2 consisted of DNA from 81 consecutive CRC Latvian patients registered at the DNA bank of the Riga Stradiņš University (mean age of diagnosis: 65 years) and DNA from 81 unselected Latvian newborns was used as a control sample.
 Group 3 consisted of DNA from 123 consecutive CRC Lithuanian patients registered at the DNA bank of the Vilnius University (mean age of diagnosis: 66 years, with missing age of diagnosis for 12 patients) and DNA from 123 unselected Lithuanian newborns was used as a control sample.
 Group 4 consisted of 795 consecutive CRC patients who underwent surgery from 1996 to 2000 in two Polish clinical hospitals: Szczecin (*n* = 550) and Bydgoszcz (*n* = 245). The mean age of diagnosis was 63 years. The control group included 795 healthy individuals matched by gender, age, and cancer family history within first-degree relatives (86 had colon cancer within first-degree relatives, 227 had other cancers, and 482 had a negative cancer family history).

The unselected newborns, used as controls in Groups 2 and 3, cannot be matched for age as in the case of the controls for Groups 1 and 4. That is, although they have no relationship with the CRC cases, it cannot be disclosed that some of them will develop CRC in the future, as they grow up. This situation decreases the statistical power of the study, because it is more difficult to identify true differences between cases and controls. Thus, we are increasing the risk of false negatives (Type II error), but on the other hand we are decreasing the risk of false positives (Type I error). In other words, while nonsignificant differences calculated for Groups 2 and 3 may be due to lack of statistical power, significant differences can only have *P* values equal to or lower than calculated.

In all cases, peripheral blood samples were collected from the patients or controls after obtaining informed consent for genetic analysis. DNA was extracted directly from leukocytes following standard methodology [15].

The study was approved by the institutional review board of ethics of the Pomeranian Medical University (Poland).

## 3. Methods

### 3.1. Genotyping

The DNA samples were genotyped using TaqMan SNP Genotyping Assays (Applied Biosystems Inc., Foster City, CA, USA) and LightCycler 480 Probes Master (Roche Diagnostics Inc., Rotkreuz, Switzerland) according to the manufacturers' recommended protocols.

### 3.2. Statistical Analysis

Differences in the genotype distribution for cases and controls between countries were analyzed applying Pearson's Chi-squared test for 18 conditions (18 genotypes) and 4 groups (4 countries), that is, 51 degrees of freedom, each.

The mode of inheritance of the phenotype associated to the risk markers is usually not part of a GWAS analysis, but the presence of a single risk allele does not necessarily increase the disease risk (e.g., if the inheritance model is recessive). For each country, each of the analyzed markers was therefore analyzed separately for its most probable inheritance model. For simplicity, only two basic inheritance models were taken into account, recessive and dominant. According to the model chosen, the presence or absence of a risk genotype was assessed for each individual. Other models such as codominant, additive, and overdominant were intentionally left out of scope to avoid compromising unnecessarily the statistical power of the study.

The particular influence of each of the genotypes of the 6 markers on disease risk was calculated by logistic regression, taking the inheritance model into account and independently for each country. Sex or age was not available for the Latvian and Lithuanian sample and could not be systematically adjusted; therefore, we chose an unconditional regression model to analyze them. In contrast, the Estonian and the Polish samples had paired controls matched for sex and age. In these two cases, a conditional regression model was preferred. Bonferroni correction for multiple testing was applied in all cases since two different inheritance models were put to the test for each SNP and country.

Cumulative effects were explored by a similar approach, but this time focusing on the number of cumulated risk genotypes (again taking the inheritance model into consideration) for each individual. For each country, a list was generated with all risk markers sorted by increasing *P* value (calculated as described above). Using these lists as a basis, ORs were calculated for compound carriers of different numbers of risk markers (following the order of the list). Here again, Bonferroni correction for multiple testing was applied, since there were five checked pools of risk markers for each country.

There were three choices for establishing the reference to calculate said ORs. One was to take the group of noncarriers as a reference, but frequently the size of that group was small (in one case even inexistent), and could therefore account for artificially high ORs. Another one was to take the most frequent group among controls, but that was a different group for each case, thus shifting the ORs curves up and down and making comparisons between countries and between different sizes of risk marker lists, virtually impossible. We decided to compare the observed proportion to the expected proportion (1 : 1) for the same sample size. In this way, all depicted ORs are directly comparable. The drawback is that this happens at the cost of some statistical power for the groups with larger numbers of cumulated risk markers (we make comparisons for smaller total sample sizes compared to what we would do with any other method). This method is very conservative and does not increase the risk of type I error, rather the opposite, but since the present study is an exploratory one, we were concerned more about false positives than about false negatives.

All calculations were done in R, version 2.15.2 [16].

## 4. Results

### 4.1. Genotypes

Genotyping success rate was 100% for all 6 SNPs. There was a significant deviation from Hardy-Weinberg equilibrium for rs4464148 among cases in the Lithuanian sample (*P* = 0.045); however, a deviation in the group of cases is not unexpected, since this group is not a representative population sample.

The genotype distribution shows some divergences between samples. As shown in Table 1, there are differences greater than 12 percentage points between countries: for example, in the Latvian sample, 49.4% of controls had genotype AC for rs3802842, while in the Lithuanian sample there were 36.7% (actually closer to the percentage observed among cases in the Latvian sample, 33.3%). Other remarkable differences, larger than 12 percentage points, affected cases from Latvia and Lithuania for rs4779584 (genotype CC), rs3802842 (genotypes AA and AC), rs4464148 (genotype TC), and rs4939827 (genotypes CT and TT). The Latvian sample also diverged largely from the Estonian sample among controls for rs6983267 (genotype TT, ~10 percentage points) and rs4939827 (genotype CT, ~13 percentage points).

Still, these differences between countries were not statistically significant, neither in the control group (Pearson's Chi-squared test, *P* = 0.98, df = 51) nor in the group of cases (Pearson's Chi-squared test, *P* = 0.95, df = 51). Analyzing each SNP separately does not change the situation; the lowest *P* value does not show any significant difference between countries (Pearson's Chi-squared test, *P* = 0.12, df = 6, for rs6983267 among controls).

### 4.2. Inheritance Models

**Figure 1:**
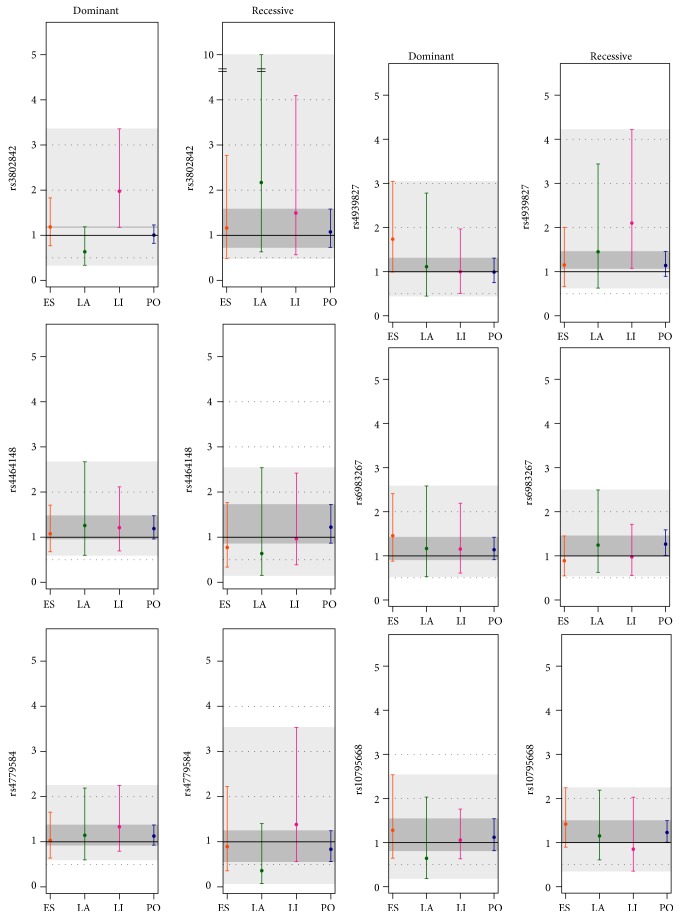
Disease risk for each inheritance model, country, and marker. Disease risk is shown in the *y*-axis as OR (circles) with 95% confidence intervals. Overlapping regions of the confidence intervals are shown in dark grey; not fully overlapping regions are shown in light grey. Note the discontinuous *y*-axis for the recessive model in rs380284.

A general overview of the estimated disease risk (in OR and 95% CI) depending on the inheritance model and country for each of the analyzed SNPs is presented in Figure 1. There is an overlapping region of the 95% CI for each SNP and inheritance model for all countries, as expected from the fact that there were no significant differences in the genotype distribution. However, it can be seen that country and inheritance model have both a visible effect on the estimated disease risk. Exemplary, it can be seen for rs4464148 an OR similar for all countries for the dominant model (and all ORs are within the overlapping region of the 95% CI for all countries), but for the recessive model, Estonia, Latvia, and Lithuania have an estimated decrease in risk, opposite to Poland.

For each country and marker, the inheritance model is chosen that maximizes disease risk for the given risk allele. Only the markers rs4779584 and rs4464148 share the same inheritance model for all countries (Table 2). The country-specific analysis of the separate effects of each marker showed a marginal association for the markers rs6983267 and rs10795668 in the Polish sample and for marker rs4939827 in the Lithuanian sample. However, after applying correction for multiple testing, these associations were not significant any more. In contrast, marker rs3802842 did withstand the correction for multiple testing for the Lithuanian sample (corrected *P* value = 0.022).

Interestingly, the analysis of the linear cumulative model, where the amount (quantitative, discrete) of risk genotypes carried by each individual was taken as an independent variable, showed a statistically significant association that withstood the correction for multiple testing, for all samples (Estonia: corrected *P* value = 0.018; Lithuania: corrected *P* value = 0.0034; Poland: corrected *P* value = 0.0076) except for the Latvian one (nominal *P* value = 0.137), which was the smallest sample of all four (Table 2).

Knowing that the linear cumulative model was effectively explaining the observed data gave us the needed support to proceed to the next analysis step, where we tried to determine the cumulative effects of a particular amount of cumulated risk markers from a sorted list out of the 6 markers analyzed (see Section 2).

**Figure 2:**
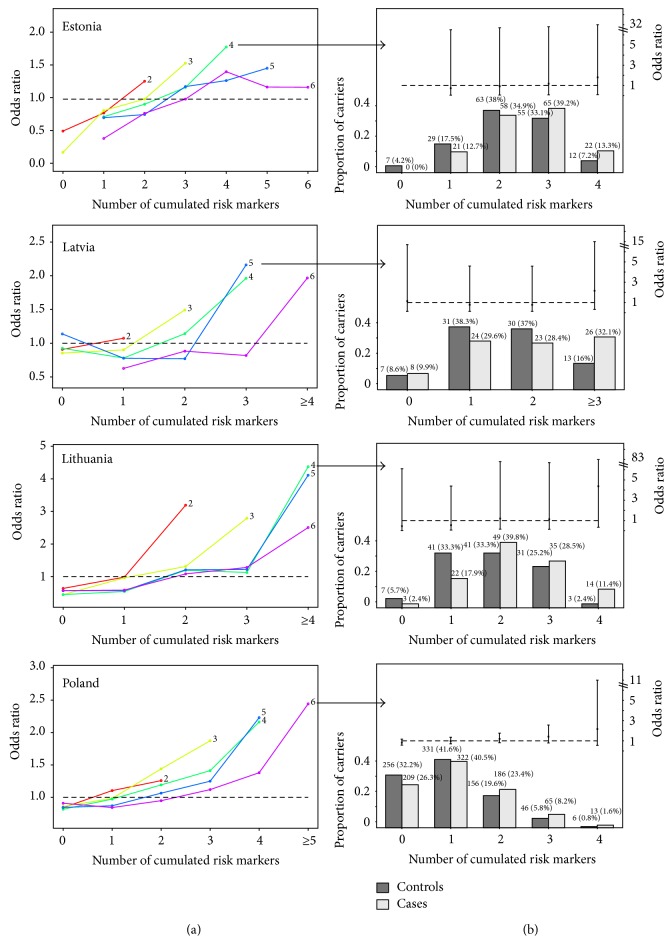
The association between colorectal cancer risk (*y*-axis) and the number of cumulated risk markers carried by a single subject (*x*-axis) is depicted at (a), independently for each country. The numbers attached to the end of each curve stand for the pool size of markers out of which the number of cumulated risk markers is calculated (see Section 2 for more details). The curve reaching the highest odds ratio (arrow) is represented in detail with confidence intervals and frequency histograms at (b). Note that the pool size of markers may be larger than the number of cumulated risk markers carried by a single subject. The odds ratio for 0 cumulated markers could not be calculated for the Estonian sample due to the complete absence of noncarriers among cases.

Taking these country-specific lists as a basis, with markers sorted by increasing *P* value, we analyzed the influence of the number of cumulated markers on the risk of CRC. Disease risk was calculated for compound carriers of risk markers, separately for each country. Reference was the expected proportion compound carriers among cases and controls (1 : 1 in all cases). The curves depicting that relationship were systematically drawn for an increasing pool of markers out of which the number of cumulated markers was withdrawn: there is a curve showing that relationship for the first two markers of the list, a different curve for the first three markers, and so on for all six markers.  Figure 2(a) shows these data separately for each country.

In all cases, the shapes of the curves ideally support the hypothesis of the cumulative model, where disease risk increases with the number of cumulated risk markers. Still, some curves are steeper than others (leading to higher odds ratios) and those are not necessarily the ones corresponding to the largest pool sizes. Only in the case of Poland, the highest odds ratio is reached for the largest pool of markers (all six markers analyzed).

The curve showing the highest risk for the lowest number of cumulated markers was represented again in detail (Figure 2(b)), with confidence intervals and a histogram depicting the proportion of cases and controls carrying those particular markers.

In the Estonian sample, the cumulative model for the pool of four markers reached OR 1.81 for an accumulation of all four markers. Analogously, the Latvian sample reached OR 2.16 for an accumulation of three or more risk markers for the pool of five markers. The Lithuanian sample achieved an OR of 4.37 for an accumulation of all four markers out of a pool of four. The Polish sample reached OR 2.16 for an accumulation of four markers out of a pool of four. However, as noticeable from the broad confidence intervals at each position, none of the differences in disease risk for neither of the samples was statistically significant for any of the possible marker pool sizes.

## 5. Discussion

In this study 6 SNPs were analyzed that, according to previous literature data, could be low-risk genetic markers for CRC. 1165 consecutive CRC cases and 1165 controls from Estonia, Latvia, Lithuania, and Poland were examined to assess whether these genetic variants are significantly associated with the occurrence of colon cancer in the Eastern Baltic States and Poland and whether any similarities or differences between the populations could be identified.

Comparison of the genotyping data from Poland and the Eastern Baltic states revealed some heterogeneity; however, differences were not statistically significant. With the exception of rs4779584 and rs4664148 (both are dominant), the best suiting inheritance model, defined as the model showing the lowest *P* value, for the rest of the markers was not consistent throughout the different countries (Table 2). Although previous studies on low-risk susceptibility genes had shown population-specific effects, these affected populations from geographically distant regions [17–19]. Here, rather similar inheritance models were expected because there were earlier data showing large genetic similarities between these neighboring populations, like that for the case of the mismatch repair genes [20] or the* BRCA1* gene [21–24].

That heterogeneity in the inheritance models made a country-specific analysis more advisable than a pooled analysis, but at the cost of a loss of statistical power due to smaller sample sizes. A logistic regression analysis of each of the six markers, independently for each country, revealed a statistically significant association between a single marker and CRC risk, only for rs3802842 in the Lithuanian sample (dominant inheritance model: OR = 1.98, 95% CI = 1.17–3.36, and corrected *P* value = 0.022). But most importantly, all cumulative models, with the exception of the Latvian sample (the smallest one), showed a significant increase in the risk of developing CRC for an increasing number of cumulated markers (corrected *P* value = 0.018 for the Estonian, corrected *P* value = 0.0034 for the Lithuanian, and corrected *P* value = 0.0076 for the Polish sample).

Having demonstrated the cumulative effect of these six low-risk markers, we focused on particular combinations of markers that could be maximizing disease risk. Assuming that some markers would play a larger role than others, a country-specific list of markers was created, ordered by increasing *P* value for an association with CRC risk. The cumulative effect was then tested for each pool size. Some of the marker combinations showed high odds ratios (up to 4.37 in the Lithuanian sample), but none of these differences was statistically significant.

To summarize, the present study demonstrated significant cumulative effects for the total of the 6 analyzed markers but failed to show significant effects of particular combinations assuming particular inheritance models.

Still, it is worth to mention some advantages shown by the proposed stepwise approach in comparison with previous analyses of cumulative effects where there is no focused analysis of ordered pools of markers [14]. Exemplary, from Figure 2 we can learn that maximizing the marker pool may lead to a relative decrease of disease risk (e.g., the pool of 4 markers has a higher odds ratio for the Estonian sample than the pools of 5 or six markers), as expected from the fact that some markers do not seem to have a large effect on disease risk (Tables 1 and 2), and, in the absence of interaction effects, may only lead to a decrease in the sensitivity and the power of the study if included in the model.

Further studies should include larger sample sizes and country-specific sets of genetic markers to create more accurate cumulative models before they could be applied in clinical practice.

## Figures and Tables

**Table 1:** Genotype frequencies for SNPs rs3802842, rs4464148, rs4779584, rs4939827, rs6983267, and rs10795668 among cases and controls in Estonia, Latvia, Lithuania, and Poland.

Marker	Risk allele	Genotypes	Estonia				Latvia				Lithuania				Poland			
			Cases		Controls		Cases		Controls		Cases		Controls		Cases		Controls	
		AA	87	52,4%	93	56,0%	46	56,8%	37	45,68%	50	40,7%	70	56,9%	408	51,3%	411	51,7%
rs3802842	C	AC	66	39,8%	61	36,7%	27	33,3%	40	49,38%	62	50,4%	45	36,6%	330	41,5%	331	41,6%
		CC	13	7,8%	12	7,2%	8	9,9%	4	4,94%	11	8,9%	8	6,5%	57	7,2%	53	6,7%

		TT	77	46,4%	87	52,4%	35	43,2%	40	49,38%	58	47,2%	63	51,2%	337	42,4%	371	46,7%
rs4464148	C	TC	74	44,6%	62	37,3%	41	50,6%	35	43,21%	45	36,6%	47	38,2%	357	44,9%	346	43,5%
		CC	15	9,0%	17	10,2%	5	6,2%	6	7,41%	20	16,3%	13	10,6%	101	12,7%	78	9,8%

		CC	99	59,6%	97	58,4%	49	60,5%	52	64,20%	58	47,2%	70	56,9%	446	56,1%	467	58,7%
rs4779584	T	CT	58	34,9%	59	35,5%	29	35,8%	22	27,16%	53	43,1%	44	35,8%	301	37,9%	272	34,2%
		TT	9	5,4%	10	6,0%	3	3,7%	7	8,64%	12	9,8%	9	7,3%	48	6,0%	56	7,0%

		CC	32	19,3%	50	30,1%	15	18,5%	19	23,46%	25	20,3%	27	22,0%	157	19,7%	167	21,0%
rs4939827	T	CT	87	52,4%	71	42,8%	46	56,8%	45	55,56%	52	42,3%	69	56,1%	393	49,4%	416	52,3%
		TT	47	28,3%	45	27,1%	20	24,7%	17	20,99%	46	37,4%	27	22,0%	245	30,8%	212	26,7%

		TT	37	22,3%	51	30,7%	16	19,8%	17	20,99%	26	21,1%	28	22,8%	190	23,9%	209	26,3%
rs6983267	G	TG	90	54,2%	73	44,0%	38	46,9%	41	50,62%	63	51,2%	61	49,6%	392	49,3%	410	51,6%
		GG	39	23,5%	42	25,3%	27	33,3%	23	28,40%	34	27,6%	34	27,6%	213	26,8%	176	22,1%

		AA	17	10,2%	21	12,7%	9	11,1%	5	6,17%	12	9,8%	13	10,6%	88	11,1%	96	12,1%
rs10795668	A	AG	71	42,8%	81	48,8%	33	40,7%	40	49,38%	59	48,0%	56	45,5%	325	40,9%	360	45,3%
		GG	78	47,0%	64	38,6%	39	48,1%	36	44,44%	52	42,3%	54	43,9%	382	48,1%	339	42,6%

**Table 2:** Genotype frequency analysis for each of the studied 6 risk markers, divided by country. ^1^Conditional, ^2^Unconditional logistic regression. ^#^After Bonferroni correction for multiple testing.

Marker	Risk allele	Estonia		Latvia		Lithuania		Poland	
		Inheritance model	*P* value^1^ OR (95% CI)	Inheritance model	*P* value^2^ OR (95% CI)	Inheritance model	*P* value^2^ OR (95% CI)	Inheritance model	*P* value^1^ OR (95% CI)
rs3802842	C	Dominant	0.45 1.18 (0.77–1.83)	Recessive	0.23 2.17 (0.63–8.64)	Dominant	*0.011 *(*0.022*)^#^ 1.98 (1.17–3.36)	Recessive	0.72 1.07 (0.73–1.58)

rs4464148	C	Dominant	0.76 1.08 (0.68–1.71)	Dominant	0.55 1.26 (0.60–2.67)	Dominant	0.52 1.20 (0.69–2.08)	Dominant	0.11 1.19 (0.96–1.47)

rs4779584	T	Dominant	0.91 1.03 (0.64–1.66)	Dominant	0.68 1.14 (0.60–2.19)	Dominant	0.28 1.33 (0.79–2.25)	Dominant	0.24 1.13 (0.93–1.37)

rs4939827	T	Dominant	0.054 1.74 (0.99–3.05)	Recessive	0.39 1.45 (0.63–3.44)	Recessive	*0.033* (0.066)^#^ 2.10 (1.07–4.22)	Recessive	0.29 1.14 (0.89–1.46)

rs6983267	G	Dominant	0.14 1.46 (0.88–2.41)	Recessive	0.54 1.24 (0.62–2.49)	Dominant	0.66 1.15 (0.61–2.19)	Recessive	*0.043* (0.086)^#^ 1.23 (1.01–1.59)

rs10795668	A	Recessive	0.13 1.42 (0.90–2.24)	Recessive	0.66 1.15 (0.61–2.19)	Dominant	0.84 1.06 (0.63–1.76)	Recessive	*0.04 *(0.08)^#^ 1.23 (1.01–1.50)

all		Cumulative	*P* = *0.0089* (*P = 0.018*)^#^	Cumulative	*P* = 0.137	Cumulative	*P* = *0.0017* (*P = 0.0034*)^#^	Cumulative	*P* = *0.00038* (*P = 0.0076*)^#^
